# Annual Meetings of Hospitals

**Published:** 1921-05-21

**Authors:** 


### ANNUAL MEETINGS OF HOSPITALS.

### Shropshire Association for the Prevention of Consumption.

The annual general meeting was held at Shrewsbury on Saturday, April 23, Lord Kenyon presiding. Reports on the work carried on at the King Edward VII. Sanatorium at Shirlett, near Much Wenlock, were presented. The average length of stay of the patients was 151 days; the average weekly cost of maintenance was £2 2s. 7d. per patient, and the administrative expenses worked out at about 5 per cent. of the total. Developments reported during the year included the establishment of a sanatorium workshop, by the aid of which many useful repairs had been carried out by the patients themselves, thus reducing the cost. The least satisfactory feature of the report was the announcement that there was a bank deficit of £298, which was attributed to a falling off in subscriptions. Lord Kenyon, moving the adoption of the report, said the Association had cause for satisfaction at the results of its ten years' work. He paid a special tribute to the work of Dr. Turner, the house-surgeon, and of Dr. Elliott, who acted as house-surgeon during the interval that preceded the appointment of Dr. Turner. The services of Miss Steaines as matron since the opening of the sanatorium ten years ago were also warmly commended. On the subject of after-care, Lord Kenyon said the importance of having strong and active local committees could not be exaggerated. Without after-care treatment of the disease was of little use. For the benefit of patients who were unable to resume their old occupations he thought an effort should be made to provide suitable light employment.

### The Elizabeth Garrett Anderson Hospital.

Miss Irene Vanbrugh presided at the annual meeting. The total income amounted to £21,520, and exceeded the expenditure by £1,707. The improvement is attributed to the receipt of an emergency grant of £1,250 from the King's Fund, but the Committee point out that the present year started with very little in current account, for running expenses and "sundry creditors" amounted to £2,760. Securities of the nominal value of £5,623, sold at a critical period to meet running expenses, realised only £2,895. Patients' fees amounted to £5,058. The Memorial Appeal Fund was closed early in the year, as it was reported that the sum required, £50,000, was assured. The beds provided by the Fund are named by means of stained-glass plaques, the work of Mr. Christopher Webb. At the end of 1919 the upper part of 134 Euston Road, presented to the hospital by Miss Aldrich-Blake, was in full use as additional accommodation for nurses and students, and the ground and basement floors were opened on May 10, 1920, as an extension of the out-patients' department. A small library, organised and managed by Miss Beatrice Harraden, had formed a noteworthy feature of the year, and was an innovation much appreciated by the patients. Gifts of books and donations are acknowledged by Miss Harraden personally. Grateful recognition was made of the beneficial activities of the Patients' League and the work of the Elizabeth Garrett Anderson Hospital Guild and the Rosa Morison House Guild.

### The Italian Hospital.

The Italian Ambassador presided at the annual meeting of the Italian Hospital. For the first time in its history the hospital had been compelled to borrow from its bankers, and started the year in debt. To meet, in some measure, the financial difficulties, a system of payment by patients was instituted. The total expenditure was £8,773, and exceeded the income by £1,057. In addition to a donation of £200, the King's Fund made an emergency grant of £400. The Festival Ball at Covent Garden Opera House was instrumental in raising over £2,000. Dr. Vincent Dickinson informed the meeting that an Italian doctor's vaccine against tuberculosis had been used in the hospital and had met with considerable success. In recognition of the great services rendered to the hospital by the Marquis and Marchioness Imperiali, when in England, the Committee have voted their Excellencies an illuminated address, which, when completed, will be despatched to Rome.

